# Case Report: A rare case of primary hepatic paraganglioma: a mimicker of hepatocellular carcinoma

**DOI:** 10.3389/fonc.2025.1570896

**Published:** 2025-09-04

**Authors:** Aitong Li, Silu Ren, Xudan Yang, Chong Yang, Tao Lu

**Affiliations:** ^1^ University of Electronic Science and Technology Hospital, Chengdu, Sichuan, China; ^2^ Sichuan Academy of Medical Sciences and Sichuan Provincial People’s Hospital, Chengdu, China

**Keywords:** hepatic paraganglioma, hepatocellular carcinoma, computed tomography, magnetic resonance imaging, case report

## Abstract

Paragangliomas (PGLs) are pheochromocytomas outside the adrenal glands, most commonly found in the retroperitoneal space, head and neck, bladder, and mediastinum. However, PGL occurring in the liver are extremely rare. We present a case of a 70-year-old woman who presented to our hospital with right upper abdominal pain, persisting for 2 years. Abdominal contrast-enhanced computed tomography (CT) revealed a 3.8 x 2.7 cm hypervascular nodule in the caudate lobe of the liver, demonstrating arterial phase hyperenhancement and portal/delayed phase washout. Magnetic resonance imaging (MRI) further demonstrated diffusion restriction and low signal intensity in the hepatobiliary phase (HBP) of the nodule. Based on these imaging features, hepatocellular carcinoma (HCC) was initially diagnosed radiologically. Surgical resection was performed, and immunohistochemical staining revealed positivity for chromogranin A (CgA), synaptophysin (Syn), and S - 100 protein, confirming the diagnosis of primary hepatic PGL (HPGL). This case highlights that hypervascular lesions with washout and HBP hypointensity may mimic HCC. Pathological verification is crucial, especially in patients without typical HCC risk factors. Although exceedingly rare, HPGL should be considered in the differential diagnosis of hypervascular hepatic nodules demonstrating typical arterial phase hyperenhancement and portal/delayed washout on CT/MRI, particularly in female patients presenting with nonspecific symptoms and lacking typical HCC risk factors such as hepatitis, alcohol abuse, or elevated tumor markers.

## Introduction

Paraganglioma (PGL) is a relatively rare neuroendocrine tumor (1). In the World Health Organization 2017 classification, pheochromocytomas (PCCs) are categorized as adrenal tumors, while PGLs are classified as extra-adrenal tumors (2). PGLs are divided into two categories: nonfunctional tumors, which produce little catecholamines, and functional tumors, which secrete excessive catecholamines, leading to symptoms such as paroxysmal hypertension, headache, palpitations, and sweating (3). PGLs are most commonly found in the retroperitoneal space (55.2%), head and neck (25.6%), bladder (5.6%), and mediastinum (3.2%) (4). However, PGLs occurring in the liver are exceedingly rare. Only eight cases of primary hepatic PGLs (HPGLs) have been reported in the English literature (4–11). However, none of these reports described the imaging features of this rare tumor in detail. This report describes another rare case of primary HPGL and its computed tomography (CT) and gadopentetate dimeglumine-enhanced magnetic resonance imaging (MRI) features, accompanied by a literature review.

## Case report

A 70-year-old woman presented to our hospital with right upper abdominal pain, persisting for 2 years. The patient had no history of viral hepatitis, blood transfusions, or alcohol abuse. She had a history of hypertension associated with occasional dizziness and headaches but had not received standardized antihypertensive therapy. On physical examination, vital signs were unremarkable, except for severe hypertension (162/111 mmHg). Laboratory tests, including complete blood count, liver and renal function tests, and tumor markers (including alpha-fetoprotein [AFP]), were all within normal ranges. Serological tests for hepatitis, including hepatitis B surface antigen (HBsAg) and anti-hepatitis C virus antibody (anti-HCV), were all negative.

**Figure 1:**
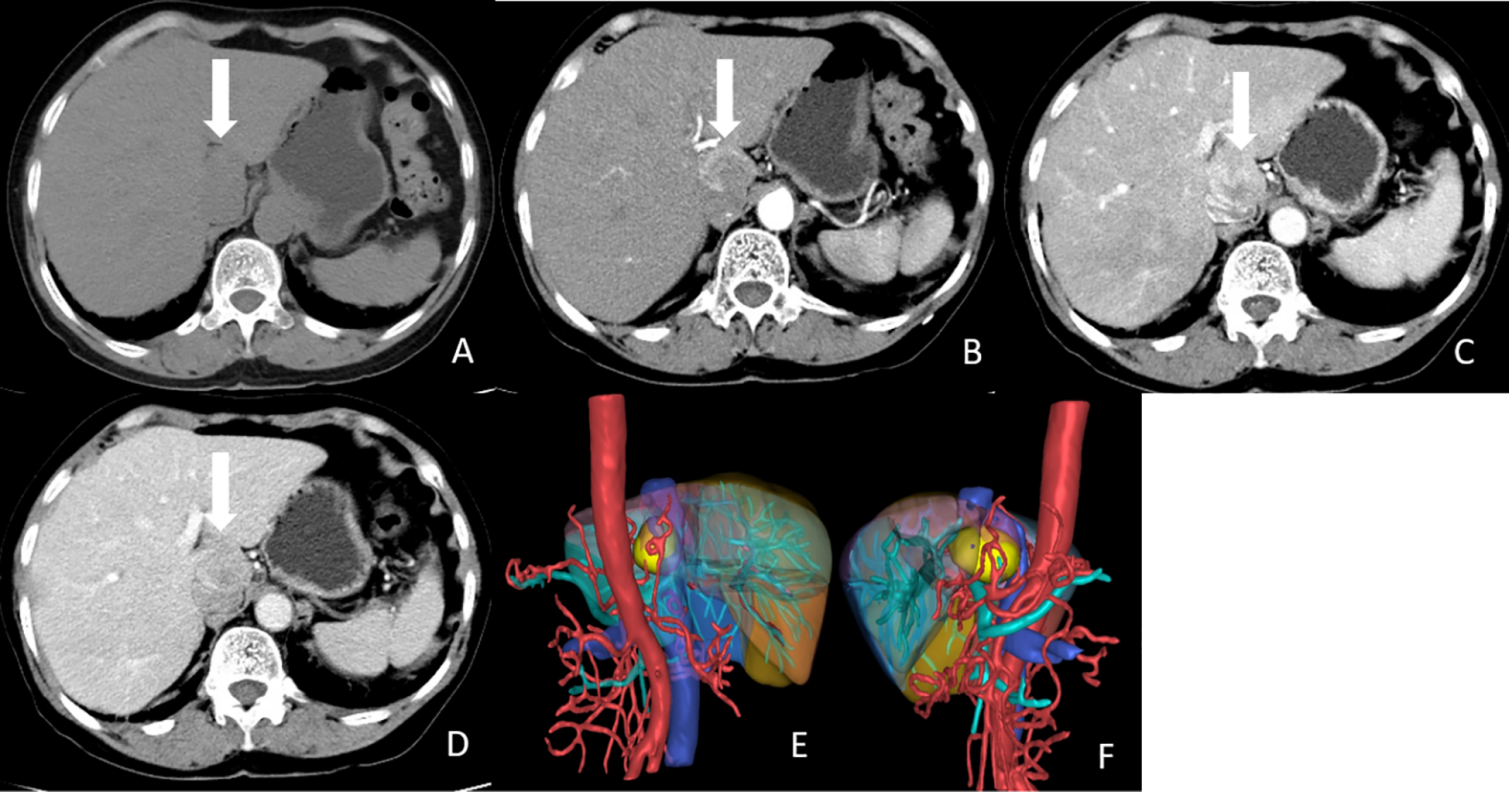
CT images of the tumor. **(A)** Non-enhanced CT image showed a hypodense, poor-marginated round nodule measured 3.8x2.7 cm in the caudate lobe of the liver (white arrow). **(B)** Axial arterial phase image showed marked heterogeneous enhancement of the nodule (white arrow). **(C)** Axial Portal phase image showed washout of this nodule. (white arrow). **(D)** Axial delayed phase image showed compression of IVC by the tumor (white arrow). **(E, F)** 3D reconstruction of CT images. The tumor was labeled with yellow, liver was labeled with brown, abdominal aorta, and hepatic artery were labeled with red, portal vein and its branches were labeled with cyan, and hepatic vein and inferior vena cava were labeled with blue.

**Figure 2:**
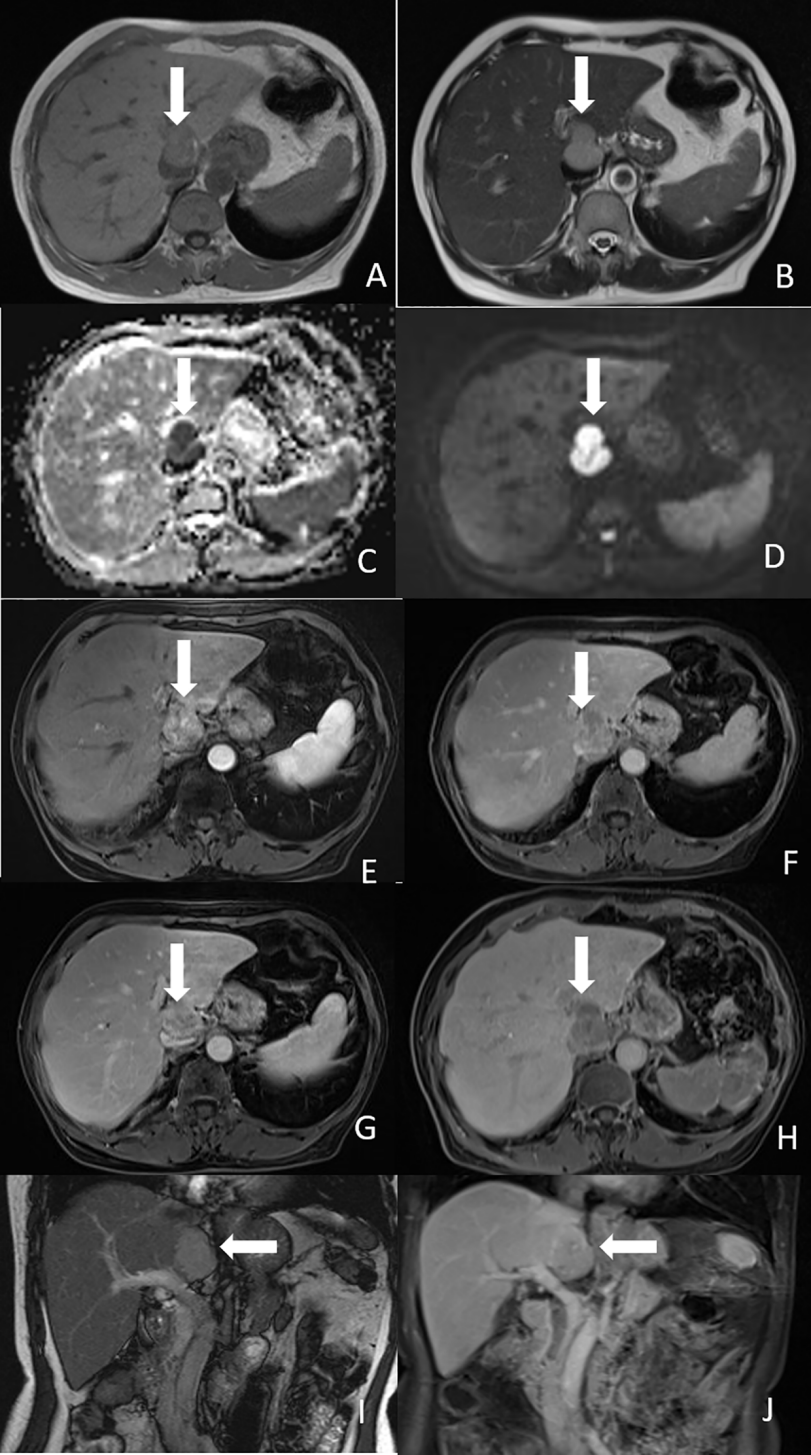
Magnetic resonance imaging of the tumor. **(A)** Axial T1-weighted image showed the tumor was hypointense (white arrow). **(B)** Axial T2-weighted image showed the tumor was mild hyperintense (white arrow). **(C)** ADC map showed low signal intensity of the tumor (white arrow). **(D)** Diffusion-weighted image showed the tumor had diffusion restriction (white arrow). **(E)** Arterial phase image showed heterogeneous enhancement of the nodule (white arrow). **(F)** Portal phase image showed heterogeneous washout of the nodule (white arrow). **(G)** Delayed phase showed compression of IVC by the tumor (white arrow). **(H)** Hepatobiliary phase image showed hypointensity of the tumor (white arrow). **(I)** Coronal T2-weighted image showed the tumor was located in the caudate lobe of the liver (white arrow). **(J)** Coronal portal phase image showed hypointensity of the tumor (white arrow).

Contrast-enhanced abdomen CT revealed a hypodense, poorly marginated round nodule measuring 3.8 x 2.7 cm in the caudate lobe of the liver, exerting a mass effect on the inferior vena cava (IVC). The nodule showed marked heterogeneous enhancement in the arterial phase following contrast injection, with washout observed in the portal and delayed phases (**Figure 1**). MRI of the abdomen revealed that the tumor was hypointense on T1-weighted imaging and mildly hyperintense on T2-weighted imaging. The nodule exhibited low signal intensity on the apparent diffusion coefficient (ADC) map, with diffusion restriction observed on diffusion-weighted imaging (DWI) (**Figures 2A–D**). After injection of gadopentetate dimeglumine, the nodule showed heterogeneous enhancement in the arterial phase and heterogeneous washout in the portal venous and delayed phases (**Figures 2E–G**). The tumor displayed low signal intensity in the hepatobiliary phase (HBP) (**Figure 2H**). Both CT and MRI suggested the possibility of hepatocellular carcinoma (HCC).

**Figure 3:**
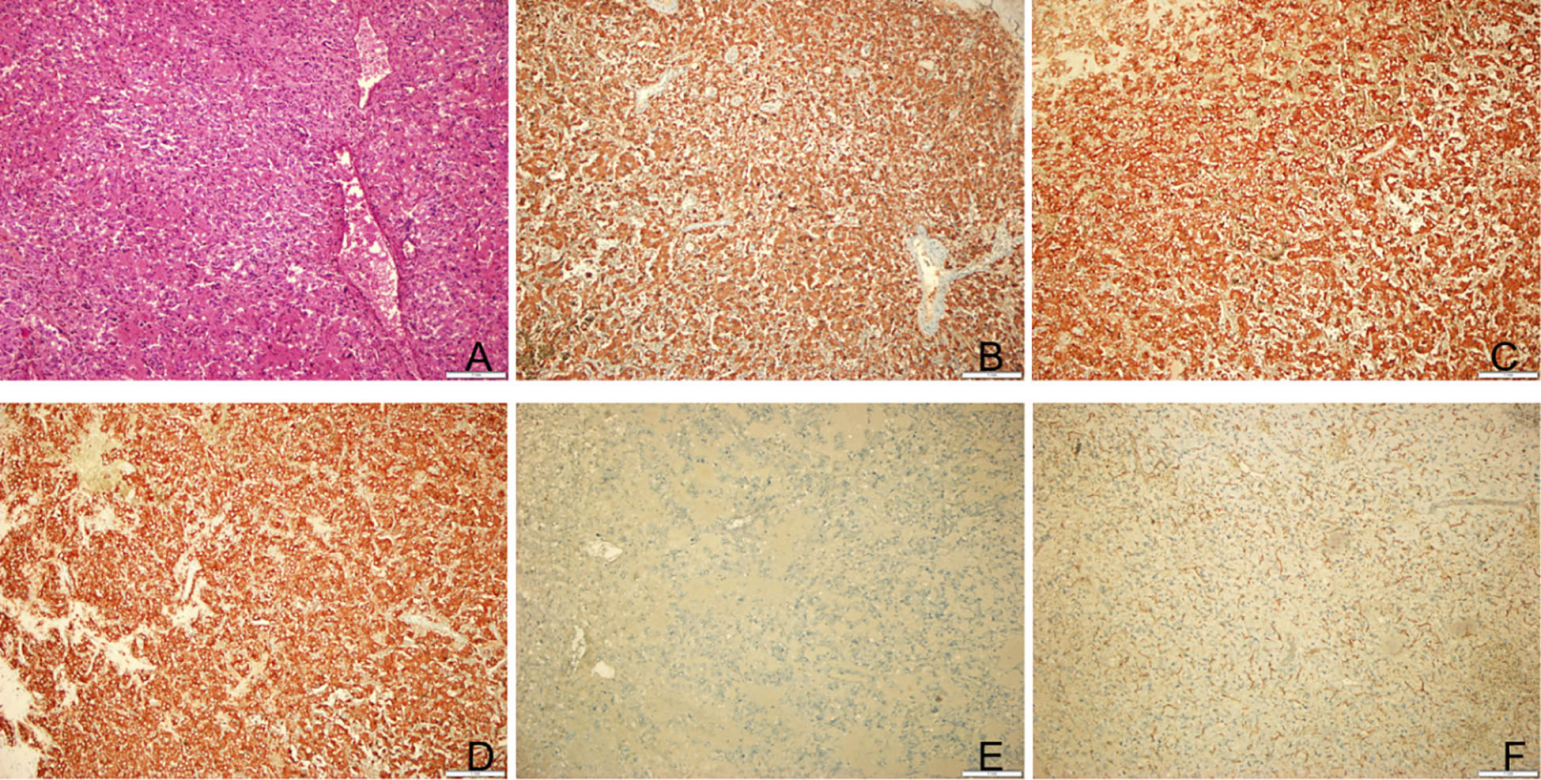
Postoperative pathological results of the tumor. **(A)** Hematoxylin-eosin staining of tumor tissue. Original magnification: 100. **(B)** Immunohistochemical staining for indicated markers. The tumor cells were positive for CgA. Original magnification: 100. **(C)** The tumor cells were positive for Syn. Original magnification: 100. **(D)** The tumor cells were positive for CD10. Original magnification: 100. **(E)** The tumor cells were positive for S100. Original magnification: 100. **(F)** The tumor cells were positive for CD34. Original magnification: 100.

**Figure 4:**
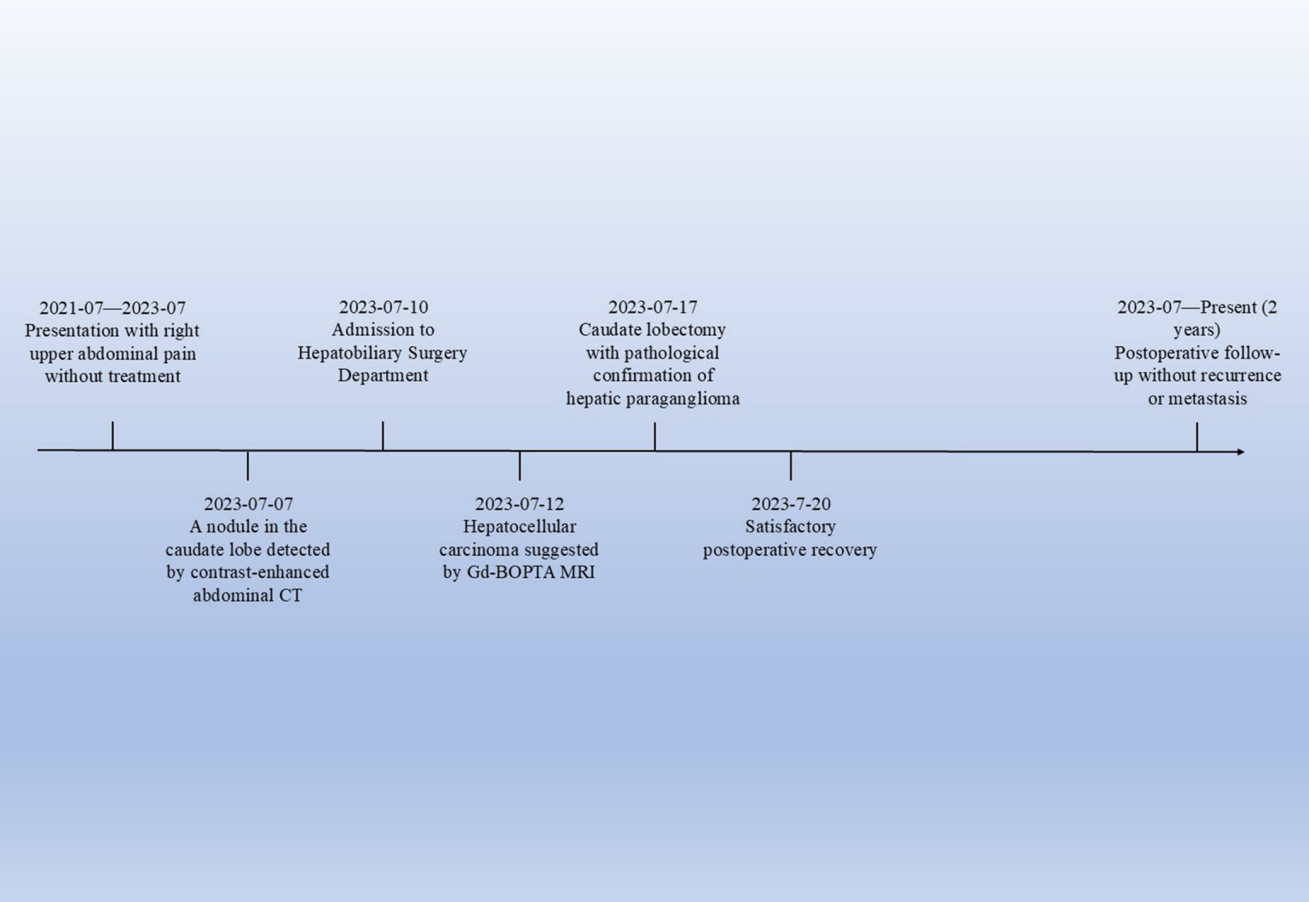
Timeline of the patient’s clinical course. This timeline illustrates the key clinical events of the patient, from the onset of symptoms to postoperative follow - up.

Subsequently, anatomical resection of the caudate lobe was performed. Although the patient lacked a history of chronic liver disease, contrast-enhanced CT and MRI showed features consistent with HCC, including arterial phase hyperenhancement, portal/delayed washout, diffusion restriction, and hypointensity in the HBP. Additionally, the patient presented with a 2-year history of recurrent right upper abdominal pain, indicating symptomatic mass effect, which established the surgical indications. During surgery, a solitary mass measuring 3.8 x 3 cm was found, with no fluctuations in blood pressure. Postoperatively, the patient’s blood pressure stabilized at 140/93 mmHg. Hematoxylin-eosin (H&E) staining of the tumor tissue revealed irregular tumor cells with pink cytoplasm and sustentacular cells surrounded by a vascular net (**Figure 3A**). Immunohistochemical staining showed that the tumor tissue was positive for CgA, Syn, S - 100, CD10, and CD34, but negative for AFP, cytokeratin 19 (CK19), CK7, GS, GPC3, HSP70, with a Ki-67 labeling index of 2% (**Figures 3B–F**). Finally, the pathological diagnosis of PGL was confirmed (**Figure 4**).

## Discussion

PCC originates in the adrenal medulla, whereas PGL arises from the sympathetic or parasympathetic nervous system outside the adrenal glands. Together, these tumors are known as PPGL (PCC and PGL) (1). PGLs are rare neuroendocrine tumors, also referred to as extra-adrenal PCCs. While they are more commonly found in the retroperitoneal space and the head and neck, extra-adrenal PGLs in the liver are extremely rare. Prior to this report, a comprehensive literature search of PubMed-indexed English publications identified only 8 cases of pathologically confirmed HPGLs. In the nine reported cases of HPGLs, including our own, seven were females, and only two were males, suggesting a female predominance. Additionally, as summarized in **Table 1**, most patients were aged 40 to 50 years. However, our patient was 70 years old, making her case older than those reported in previous studies.

**Table 1:** Clinical characteristics of patients with hepatic paraganglioma.

Reference	Age/sex	Symptoms	Hypertension	Primary diagnosis	Immunohistochemistry	Outcome
Li 2022 (4)	47/F	Dizziness	No	HCC	CgA(+)Syn(+) CD56(+)Vimentin(+)	No recurrence (1 year)
Miller 2022 (5)	54/F	Abdominal pain	Controlled hypertension (125/87mmHg)	N/A	CgA(+)Syn(+)	No recurrence (N/A)
Lin 2019 (6)	41/F	None	No	HCC	CD56(+)S-100(+)	No recurrence (6 months)
Liao 2018 (7)	49/F	None	No	HCC	CgA(+)Syn(+) CD56(+)NSE(+) S-100(+)	No recurrence (2 years)
You 2015 (8)	47/F	None	Mild hypertension (150/100mmHg)	HCC	CgA(+)NSE(+) S-100(+)	Recurrence (3 years)
Reif 1996 (9)	42/F	Palpitations, sweating and headaches	Severe hypertension (190/94mmHg)	Paraganglioma	Serotonin(+)	No recurrence (14 months)
Rimmelin 1996 (10)	24/M	Sweating	Persistent hypertension	Paraganglioma	CgA(+)Syn(+)	No recurrence (3 months)
Jaeck 1995 (11)	24/M	None	Severe hypertension (210/120mmHg)	Paraganglioma	CgA(+)Syn(+) NSE(+)	No recurrence (37 months)
Present case	70/F	Abdominal pain	Severe hypertension (162/111mmHg)	HCC	CgA(+)Syn(+) S-100(+)	No recurrence (18 months)

F, Female; M, Male; N/A, Not Available; CgA, Chromogranin A; Syn, Synaptophysin; CD56, Neural Cell Adhesion Molecule; NSE, Neuron - Specific Enolase.

PPGLs can secrete catecholamines, leading to a variety of clinical syndromes. Studies have shown that the most common symptoms of PPGLs include hypertension, followed by headache, palpitations, and sweating (12). The classic triad of headache, palpitations, and sweating is present in only 17% of patients, although its presence holds the greatest diagnostic value among all PPGLs manifestations (13). Approximately 10 - 15% of patients are asymptomatic (14). As shown in **Table 1**, the clinical manifestations of most HPGL patients were atypical. Two cases presented with abdominal pain, and four cases were asymptomatic, with three of these being detected during routine physical examinations. Only one case presented with the characteristic triad of headache, palpitations, and sweating, while another case presented with sweating, and one with dizziness. These symptoms may be associated with catecholamine hypersecretion. According to the literature, 70 - 90% of patients diagnosed with PPGL experience hypertension (15). In the nine reported HPGLs cases, six patients had hypertension, including three with severe hypertension and one with mild hypertension. Furthermore, none of the patients had a history of hepatitis or alcohol abuse, and their tumor markers were within normal limits. In our case, the patient presented with right upper abdominal pain and severe hypertension. Her clinical manifestations lacked specificity, making it difficult to suspect HPGL.

HPGLs can occur in any part of the liver but are more commonly found in the caudate lobe. Including our case, three tumors were located in the caudate lobe (**Table 2**). Tumor diameters ranged from 3.2 to 6.6 cm. Among them, six tumors measured <5 cm in diameter, while only two were > 5 cm. In our case, the tumor was located in the caudate lobe and measured less than 5 cm, which is consistent with previous studies. Furthermore, as three tumors were located in the caudate lobe, they were more likely to exert a compressive effect on blood vessels, primarily the IVC and hepatic veins. Previous reports have indicated that HPGL typically presented with low attenuation and a density greater than 10 HU (16). As PGLs are hypervascular tumors, they demonstrate marked heterogeneous enhancement in the arterial phase following contrast injection, with delayed washout in the portal venous and delayed phases (17). Abdominal MRI of PPGLs typically shows T1 hypointensity or isointensity and marked T2 hyperintensity (18). Similar to CT, the tumors exhibit avid arterial enhancement. DWI and ADC values are not particularly useful in differentiating between benign and malignant PPGLs (19). In our case, in addition to the arterial phase hyperenhancement and washout, the tumor showed diffusion restriction in DWI and hypointensity in the HBP. The CT and MRI features of the tumor resembled those of typical HCC. As the patient’s clinical manifestations were not specific, and AFP levels were normal, it was challenging to rule out the diagnosis of HCC.

**Table 2:** Imaging features of patients with hepatic paraganglioma.

Reference	Location	Size (cm)	Vascular compression	Imaging modality	Non–contrast scan	Artertial phase	Venous phase	Delayed phase
Li 2022 (4)	Spiegelian lobe	3.8x3.2	N/A	MRI	Hypointensity on T1WI;Hyperintensity on T2WI	Hyper-enhanced	Washout	N/A
Miller 2022 (5)	Caudate lobe	6.6	Yes	MRI	Hyperintensity on T2WI	Hyper-enhanced	N/A	Washout
Lin 2019 (6)	Segment VII	N/A	N/A	CT	N/A	Hyper-enhanced	Washout	Washout
Liao 2018 (7)	Segment VII Segment VIII Caudate Process	5.7x4.9	No	MRI/CT	Hypointensity on T1WI;Hyperintensity on T2WI/Hypodensity on CT	Hyper-enhanced	N/A	De -enhanced
You 2015 (8)	Segment III	3.6x3.4	N/A	CT	Hypodensity on CT	Hyper-enhanced	N/A	N/A
Reif 1996	Segment IV	4.5x3	N/A	MRI	Hyperintensity on T2WI	N/A	N/A	N/A
Rimmelin 1996 (10)	Segment VIII	5	Yes	MRI/CT	Isointensity on T1WI; Hyperintensity on T2WI/Hypodensity on CT	Hyper-enhanced	N/A	N/A
Jaeck 1995 (11)	Segment VIII	5	No	MRI/CT	N/A	Hyper-enhanced	N/A	N/A
Present case	Caudate lobe	4.5x3.9	Yes	MRI/CT	Hypointensity on T1WI; Hyperintensity on T2WI/Hypodensity on CT	Hyper-enhanced	Washout	Washout

CT, Computed Tomography; MRI, Magnetic Resonance Imaging; PET-CT, Positron Emission Tomography - Computed Tomography.

Pathological examination remains the gold standard for diagnosing PPGL. H&E staining of tumor tissue reveals that tumor cells are arranged in nests or *Zellballen*-like patterns. These cells are polygonal or oval-shaped, with abundant eosinophilic or granular cytoplasm. A capillary network is often observed surrounding the cell nests. A second cell population, sustentacular cells, can be identified at the periphery of the nests using immunohistochemical staining for S - 100 protein (20). Immunohistochemical staining plays a crucial role in diagnosing PGL, as it typically demonstrates that tumor cells are positive for CgA, Syn, NSE, and CD56 while negative for epithelial markers such as CK, EMA, and GPC3. Supporting cells surrounding tumor cells are generally positive for S - 100. The Ki-67 labeling index is typically below 3%. Studies have shown that the Ki-67 index is an independent risk factor for recurrence and metastasis in PPGL. The higher the Ki-67 labeling index, the stronger the metastatic potential, leading to a worse prognosis for patients with PPGL (21). Immunohistochemical staining of the tumor in the present case showed that the tumor tissue was negative for AFP, Glypican-3, and CK19, thus ruling out the diagnosis of HCC. In contrast, the tumor tissue was positive for CgA, Syn, and S - 100, confirming the diagnosis of HPGL. Furthermore, the patient’s Ki-67 labeling index was 2%, indicating a relatively favorable prognosis. No recurrence or metastasis has been observed during the 18-month follow-up period.

Surgical resection is the preferred treatment for HPGL. As functional PGLs can secrete catecholamines, preoperative pharmacological preparation (typically with alpha- and beta-adrenergic blockade) is crucial to prevent significant hemodynamic fluctuations during anesthesia induction, surgery, and the postoperative period, thereby minimizing life-threatening risks. In this case, the patient’s blood pressure was mildly elevated prior to surgery but remained stable throughout the procedure. Postoperative blood pressure was recorded at 140/93 mmHg with stable vital signs. Thus, no additional drug preparation was required.

Most abdominal PGLs are benign and can be cured by surgical excision. However, reports suggest that 10%-20% of cases may be malignant (4). The distinction between benign and malignant PGLs has long been debated, as pathological examination alone is often insufficient for differentiation. Postoperative follow-up is considered the most reliable method for identifying malignant HPGLs. The tumor should be classified as malignant if recurrence or metastasis occurs during the follow-up period (7). However, definitively determining malignancy may take more than 5 years of follow-up (22). The 5-year survival rate for malignant PGLs is generally low, often less than 50%. Among reported cases of HPGLs, seven patients did not experience recurrence or metastasis during a short follow-up period of less than 5 years, while one patient developed metastasis to segment 6 of the liver and the spleen 3 years after surgery. This highlights the need for prolonged follow-up.

HPGL must be differentiated primarily from HCC. Most patients with HCC have a history of viral hepatitis infection, such as hepatitis B virus or hepatitis C virus (23). They commonly present with symptoms such as right upper abdominal pain, abdominal distension, weight loss, weakness, or cirrhosis-related signs (24). Additionally, serum AFP and protein induced by vitamin K absence or antagonist-II are often elevated in HCC. However, AFP has limited sensitivity and specificity, particularly in early-stage HCC, and false elevations can occur in conditions such as active hepatic inflammation (e.g., viral hepatitis) or other liver masses like cholangiocarcinoma (25). Approximately 30% of patients with HCC exhibit normal AFP levels (26). According to the 2018 version of LI-RADS (LR), imaging features meeting LR - 5 criteria indicate definitive HCC. This requires nonrim arterial phase hyperenhancement (APHE) as a prerequisite, plus ≥1 of the following ancillary features: 1) For lesions 10 – 19 mm: nonperipheral “washout” in portal venous/delayed phases without other major malignant features; 2) For lesions 10 – 19 mm: ≥50% size increase within 6 months without other major malignant features; 3) For lesions ≥20 mm: presence of ≥1 additional major malignant feature (27). In our case, contrast-enhanced abdominal CT demonstrated a 3.8 × 2.7 cm lesion (>20 mm) with marked heterogeneous APHE, accompanied by washout during portal venous and delayed phases. Furthermore, the lesion exhibited diffusion restriction on DWI and hypointensity in the hepatobiliary phase. These collective imaging features satisfied LR - 5 criteria, leading to an initial diagnosis of HCC. However, Lin et al. reported that HPGL demonstrated diffuse homogeneous arterial phase enhancement, whereas HCC often exhibits heterogeneous APHE. This difference may be attributed to HPGL’s more uniform vascular network (*Zellballen* structure). Additionally, Miller et al. observed delayed washout in HPGL, potentially distinguishing it from the rapid washout typically seen in HCC, which may serve as a key discriminative feature. In summary, distinguishing between these two tumors based solely on imaging is challenging. Furthermore, the absence of typical catecholamine expression symptoms complicated the diagnosis. Nevertheless, HPGL should be considered in the differential diagnosis of hypervascular liver tumors, particularly in patients without a history of hepatitis infection, alcohol abuse, or abnormal tumor markers. Beyond HCC, a few other hypervascular lesions warrant brief consideration in the differential diagnosis, including 1) Hepatic hemangiomas, most hepatic hemangiomas are asymptomatic and more common in females. On non-contrast CT, they typically appear as well-defined hypodense nodules. Post-contrast, they demonstrate characteristic peripheral nodular enhancement with progressive centripetal filling and lack washout. A highly reliable diagnostic feature is their marked hyperintensity (“light bulb” sign) on T2-weighted imaging (28). In contrast, our case exhibits only mild hyperintensity on T2-weighted imaging and shows contrast washout. 2) Hepatocellular adenomas, rare benign liver tumors that occur predominantly in younger women and are strongly associated with hormonal factors such as oral contraceptive use. On contrast-enhanced CT, they typically show marked arterial hyperenhancement, followed by iso-enhancement in the portal venous and delayed phases (29). In contrast, our case demonstrates significant washout in the portal venous and delayed phases. 3) Hypervascular metastases (e.g., from neuroendocrine tumors), whose typical features include arterial hyperenhancement (often heterogeneous or rim-like), possible washout on portal venous/delayed phases, and frequent multiplicity (30). Diagnosis critically relies on identifying a known primary malignancy. In contrast, our case shows a solitary lesion without a known primary tumor.

Currently, ^123^I-MIBG imaging is useful for confirming the diagnosis, locating sites of PPGL, and evaluating metastases. Studies indicate that ^123^I-MIBG imaging has a specificity of 82%-84% for diagnosing primary or metastatic PCC or PGL (31). However, in this case, both the clinical symptoms and imaging features were nonspecific, and HPGL was not suspected preoperatively, which led to the decision not to employ ^123^I-MIBG imaging.

In conclusion, HPGL is an extremely rare tumor, typically found in female patients aged 40 – 50 years. Most patients lack specific clinical manifestations, and its imaging features are similar to those of HCC. While surgery remains the treatment of choice for HPGL, long-term follow-up is essential for assessing the efficacy of treatment and for the early detection of recurrence or metastasis.

## Data Availability

The original contributions presented in the study are included in the article/supplementary material. Further inquiries can be directed to the corresponding author.
